# Proximal Splenorenal Shunt in a Rare Renal Vein Anomaly: A Case Report

**DOI:** 10.7759/cureus.4754

**Published:** 2019-05-25

**Authors:** Pottakkat Biju, Karan Midha, Shahana Gupta, Raja Kalayarasan, Senthil Gnanasekaran

**Affiliations:** 1 Surgical Gastroenterology, Jawaharlal Institute of Postgraduate Medical Education and Research (JIPMER), Puducherry, IND; 2 Surgical Gastroenterology, Medical College & Hospital, Kolkata, IND

**Keywords:** left renal vein anomaly, ncph, proximal splenorenal shunt

## Abstract

Left renal vein (LRV) has been considered as the most suitable vein for proximal splenorenal shunt (PSRS), a commonly performed shunt for non-cirrhotic portal hypertension. Anatomical anomalies in LRV that can pose technical difficulty during shunt procedure are reported in 10% cases. We report a rare anomaly of LRV which precluded performance of standard end-to-side proximal splenorenal shunt and describe its management by performing an interposition end-to-end proximal splenorenal shunt.

A 50-year-old female presented with recurrent episodes of upper gastrointestinal bleed for five years. She was pale and had a massive splenomegaly. There were no signs of encephalopathy. Upper gastrointestinal (UGI) endoscopy revealed three columns of grade 3 esophageal varices, large fundal varices and mild portal hypertensive gastropathy. Duplex ultrasound and contrast-enhanced computed tomography (CECT) of the abdomen was suggestive of non-cirrhotic portal fibrosis. She underwent an interposition end-to-end proximal splenorenal shunt with inferior branch of left renal vein. She developed partial shunt thrombosis at follow-up of 18 months and underwent balloon angioplasty and metallic stenting of shunt. She is doing well at 24 months follow-up with no recurrence of symptoms and a patent shunt.

In conclusion, the presence of renal vein abnormalities does not preclude performance of PSRS with suitable modifications. A high index of suspicion is required to detect them preoperatively to avoid technical difficulties and to plan modifications of PSRS. Interposition end-to-end graft proximal splenorenal shunt is a valid option with good primary-assisted patency rate and clinical outcome.

## Introduction

Proximal splenorenal shunt (PSRS) is a well-established procedure for patients with non-cirrhotic portal hypertension (NCPH) with upper gastrointestinal bleeding refractory to medical therapy. PSRS is a onetime solution for variceal bleeding, massive splenomegaly, symptomatic hypersplenism and portal biliopathy [1]. Left renal vein (LRV) has been considered as the most suitable vein for shunt because of its close proximity and greater length which helps in obtaining a tension-free anastomosis. Anatomical anomalies in LRV, which can pose technical difficulty during shunt procedure, have been reported in up to 10% of cases [2]. We report a rare anomaly of LRV which precluded performance of standard end-to-side PSRS. An interposition end-to-end proximal splenorenal shunt was performed in this case.

## Case presentation

**Figure 1:**
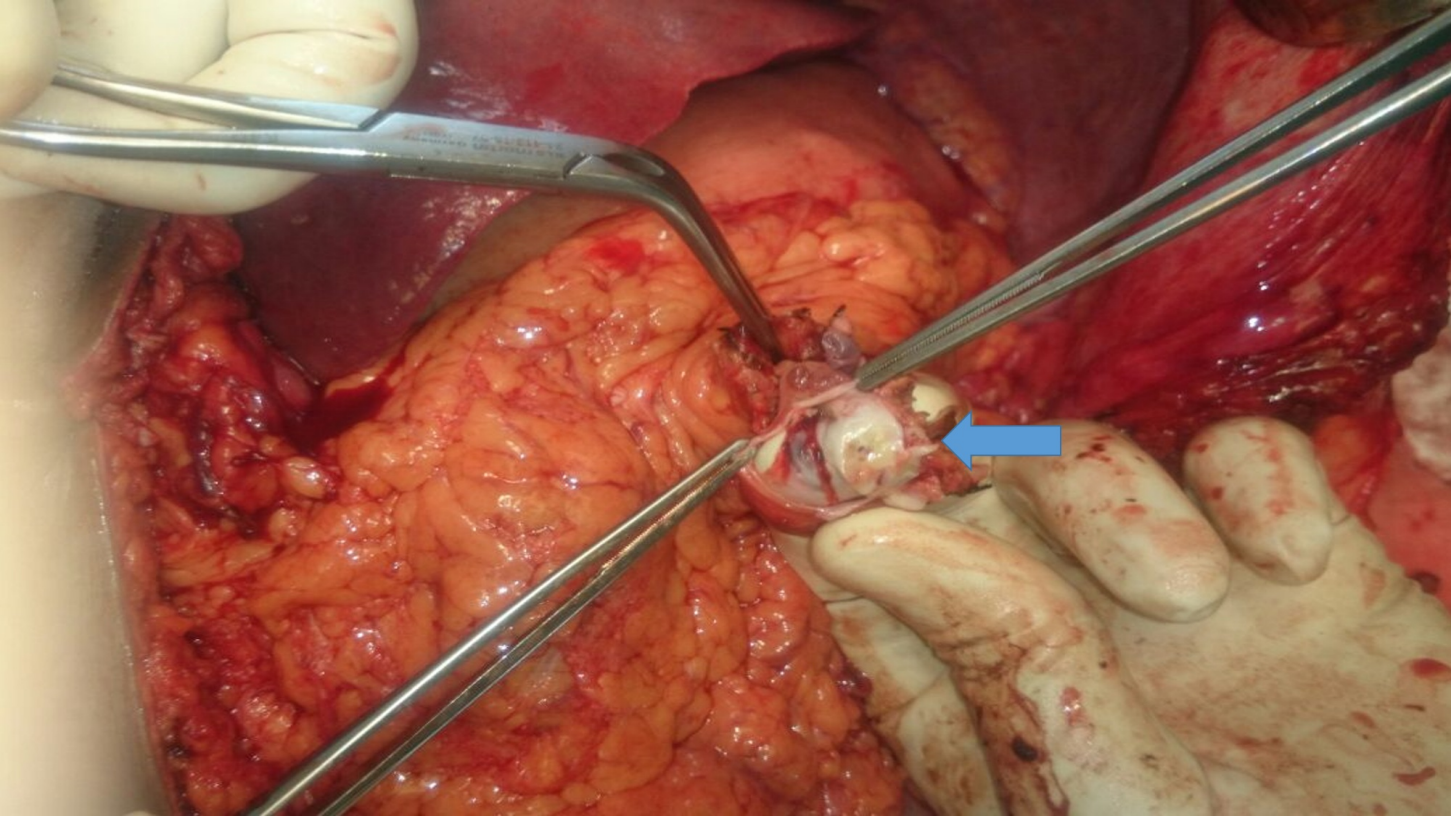
Unhealthy splenic vein wall containing atheromatous plaques (arrow).

A 50-year-old female presented with complaints of recurrent episodes of hematemesis for five years and had undergone multiple sessions of endoscopic therapies prior to admission to Department of Surgical Gastroenterology, Jawaharlal Institute of Postgraduate Medical Education and Research (JIPMER), India. She also complained of early satiety, recurrent left upper abdominal pain, easy fatigability and occasional gum bleeding. The patient never had history of jaundice, encephalopathy, abdominal distension or pedal edema. On clinical examination, she was pale, anicteric and had massive splenomegaly. She had no signs of encephalopathy. Upper gastrointestinal (UGI) endoscopy revealed three columns of large esophageal and fundal varices and mild portal hypertensive gastropathy. Duplex ultrasound and contrast-enhanced computed tomography (CECT) of the abdomen showed a normal liver and biliary system, dilated portal vein (maximum diameter - 20 mm) and splenic vein (maximum diameter - 22 mm) with no intravascular thrombus, few perisplenic and periportal collaterals. Splenomegaly was noted (23 cm in craniocaudal axis) with few infarcts. Liver function tests were normal. Blood investigations revealed hemoglobin of 7.4 g/dl with features of hypersplenism (low total leucocyte count: 1800/cubic millimeter and platelet count: 54,000/cubic millimeter). She was optimised and planned for proximal splenorenal shunt. Preoperatively, she was administered pneumococcal, H. influenzae and meningococcal vaccine. Informed consent was taken for proximal splenorenal shunt. Abdomen was opened via a left trapdoor incision. Intraoperatively no free fluid was noted in peritoneal cavity. Liver was slightly nodular. Lesser sac was opened and splenic artery was ligated after complete mobilisation of spleen. The splenic vein was then dissected at the splenic hilum before splenectomy. Around 4 cm of proximal part of splenic vein had atheromatous and calcific plaques, which was resected (Figure 1).

**Figure 2:**
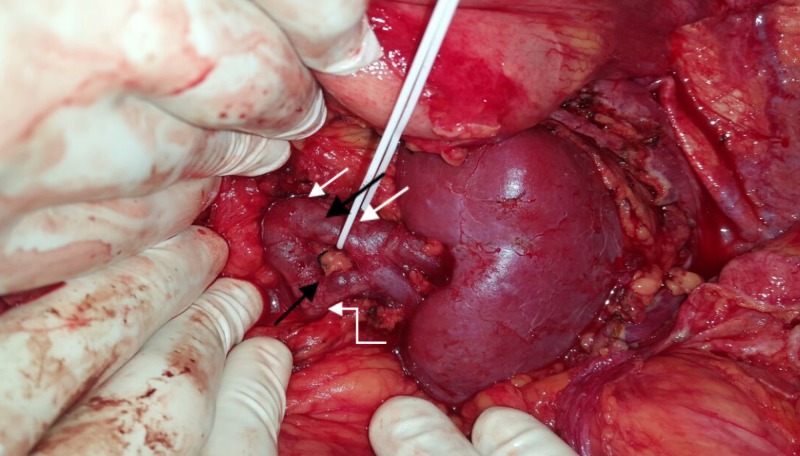
Showing the LRV anomaly – after the formation of a single vein from two tributaries at hilum of left kidney (LK), it is again dividing into two branches (superior and inferior – shown in black arrows, and superior branch being looped) and then joining again before crossing aorta (not shown). Gonadal vein (curved white arrow) was draining into the inferior branch, and adrenal vein and lumbar vein (both white arrows) were draining into superior branch. LRV: Left renal vein

**Figure 3:**
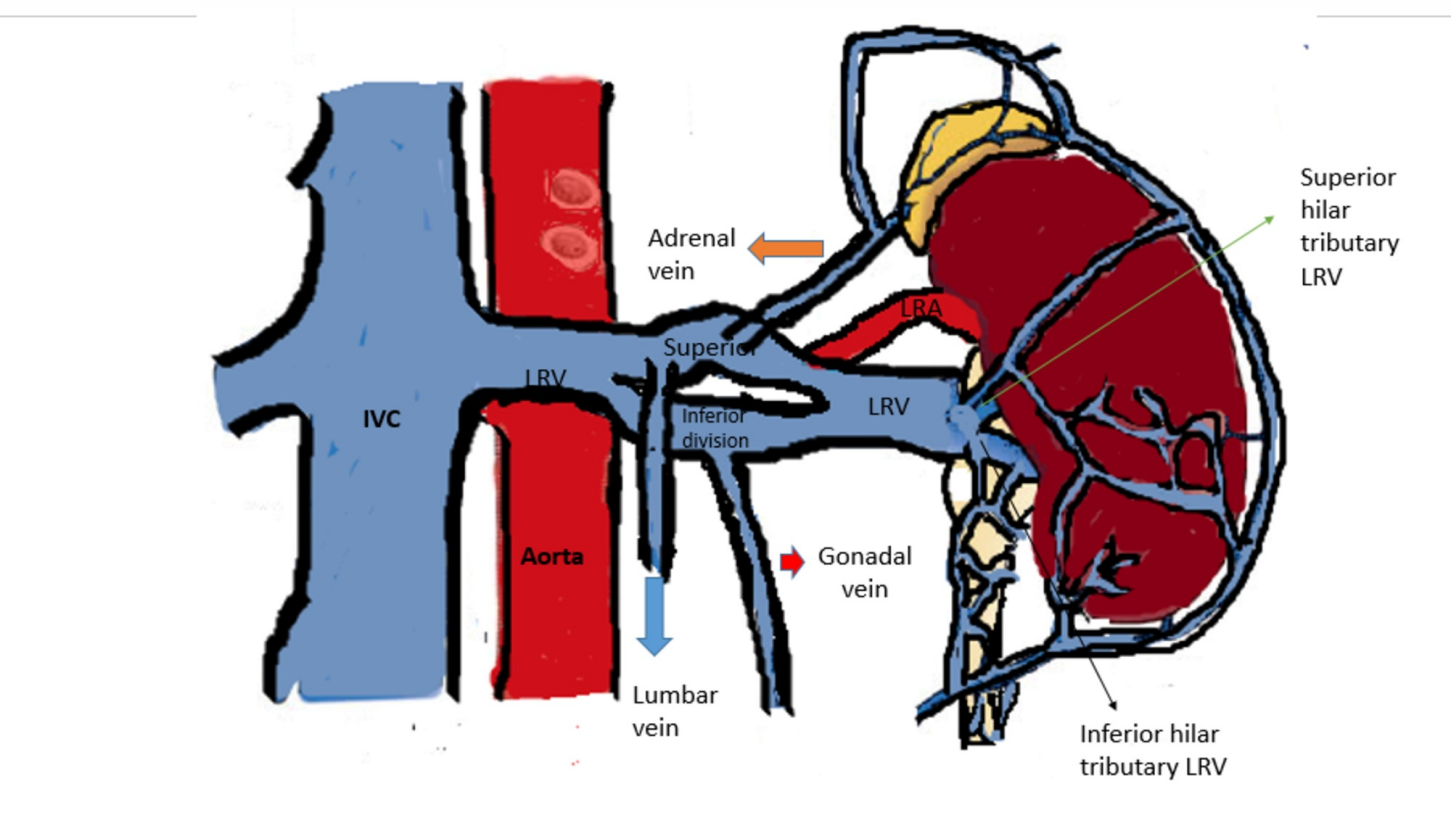
Diagram representation of our case showing the LRV anomaly – after the formation of a single vein from two tributaries at hilum of left kidney, it is again dividing into two branches (superior and inferior as labelled) and then joining again before crossing aorta. Gonadal vein (red arrow) was draining into the inferior branch, and adrenal vein (orange arrow) and lumbar vein (blue arrow) draining into superior branch. IVC: Inferior vena cava; LRV: Left renal vein; LRA: Left renal artery.

An anatomical anomaly of left renal vein was noted after formation of a single vein from two tributaries at hilum, the vein divided into two branches which again joined before crossing aorta to drain into the inferior vena cava (Figures 2, 3).

**Figure 4:**
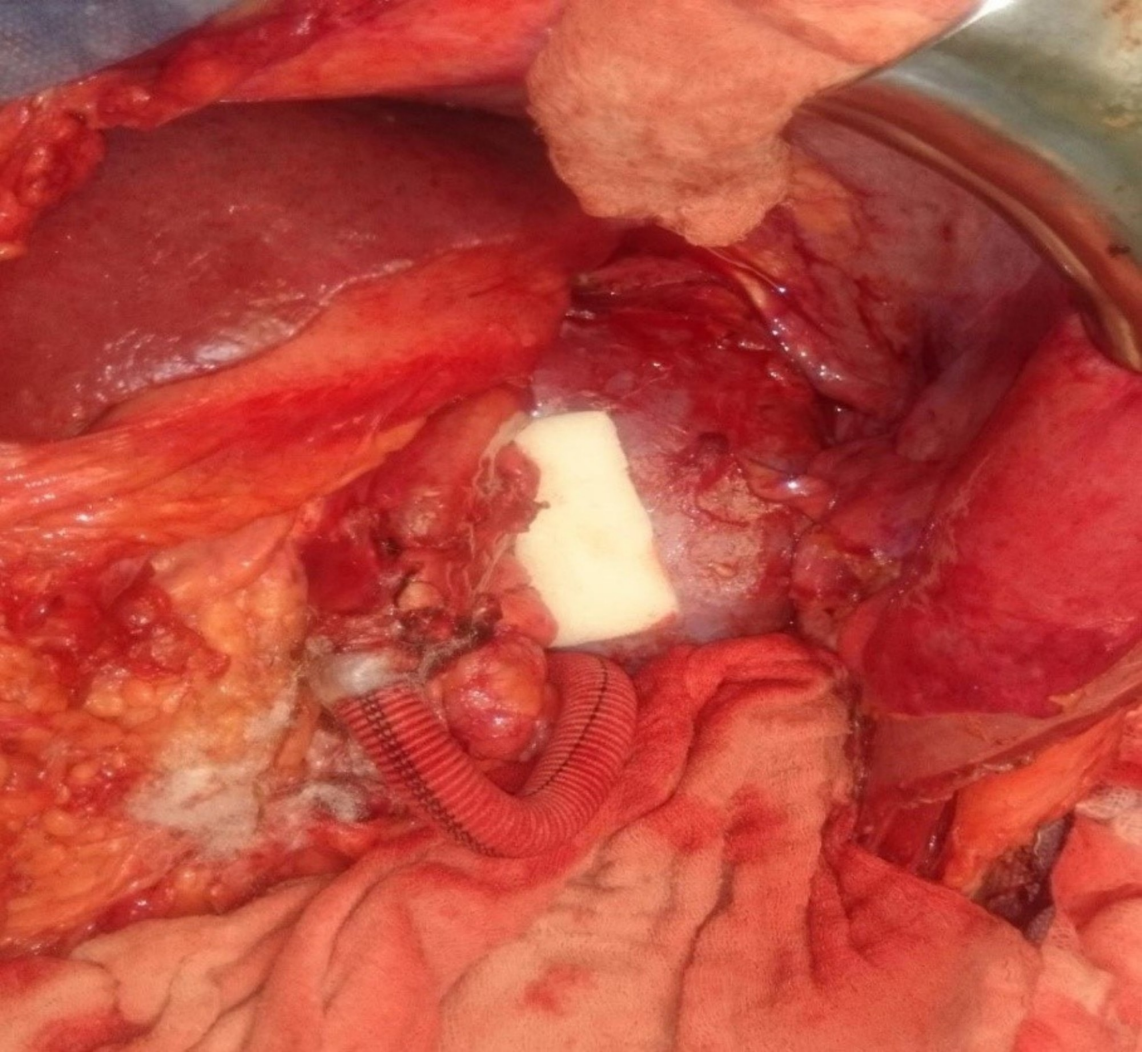
Completed interposition PSRS (8 mm ringed PTFE graft used). PSRS: Proximal splenorenal shunt; PTFE: Polytetrafluoroethylene.

Both the branches were around 10 mm in diameter. The left gonadal vein drained into the inferior branch, and left adrenal vein and lumbar veins into the superior branch. LRV was carefully dissected from the left border of aorta to the renal hilum and all the branches were looped. Interposition splenorenal shunt was planned using an 8 mm ringed polytetrafluoroethylene (PTFE) graft in view of inadequate splenic vein length. End-to-side anastomosis was planned between the graft and superior branch of left renal vein, which could not be done due to technical issues. Later the left gonadal vein was ligated and the inferior branch of left renal vein was divided and an end-to-end interposition PSRS (using an 8 mm ringed PTFE graft) was done between splenic vein and proximal part of inferior branch of left renal vein with 6-0 prolene continuous sutures (Figure 4).

**Figure 5:**
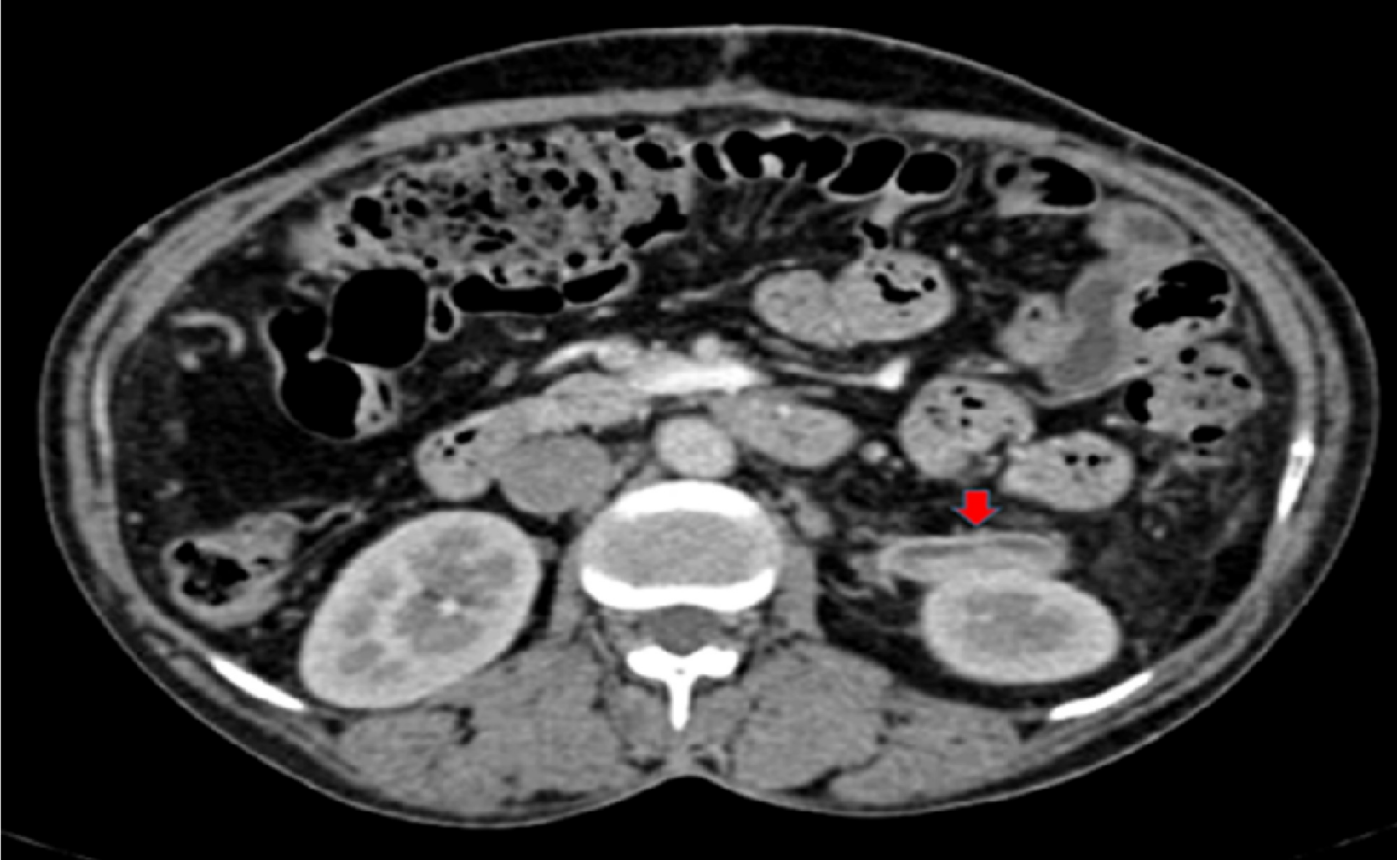
Interposition PSRS with partial thrombosis at 18 months (red arrow). PSRS: Proximal splenorenal shunt

Left kidney remained healthy and there was no congestion after the division of inferior branch of left renal vein. Left kidney mobilisation was not attempted to bring renal and splenic vein closer for anastomosis as it may have needed ureter mobilisation and resulted in floppy kidney. Core biopsy from liver was taken. Pre-shunt portal pressure was 35 mm Hg and post-shunt pressure was 22 mm Hg. The postoperative course was uneventful. Liver biopsy was suggestive of non-cirrhotic portal fibrosis (NCPF). UGI endoscopy done in the follow-up after six months showed resolution of varices and liver and renal functions as well as routine urine examination remained normal. The patient was kept on anticoagulants with a target international normalised ratio (INR) of 2.5-3.0. At 18 months follow-up, the patient developed partial shunt thrombosis demonstrable on CECT abdomen (Figure 5).

**Figure 6:**
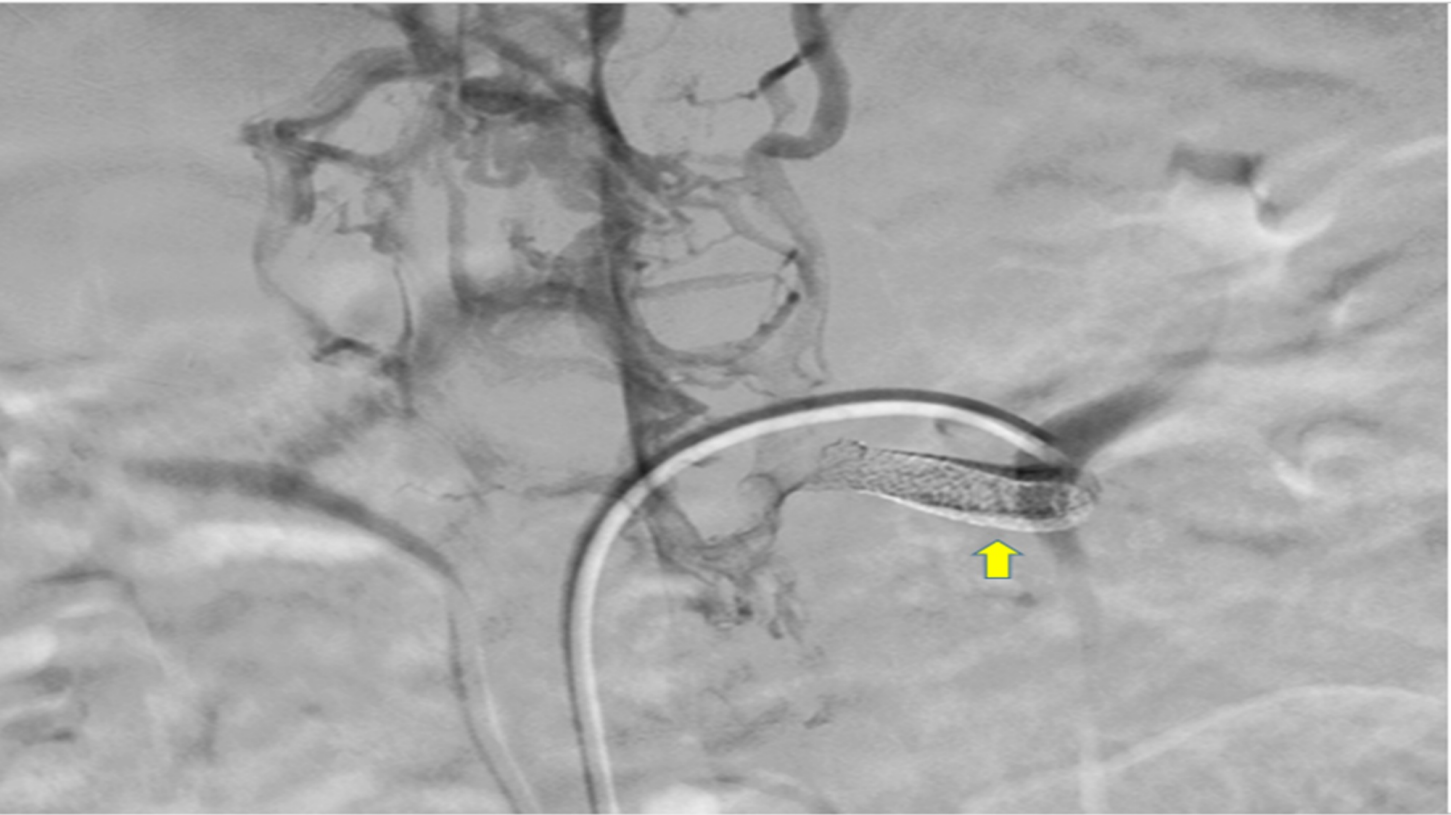
Interposition shunt post-stenting (yellow arrow).

Stenting of the thrombosed shunt was planned. Angiogram was performed which revealed 70% narrowing of the graft. Balloon angioplasty was done and an 8 mm x 37 mm bare metallic stent was deployed in the shunt (Figure 6).

**Figure 7:**
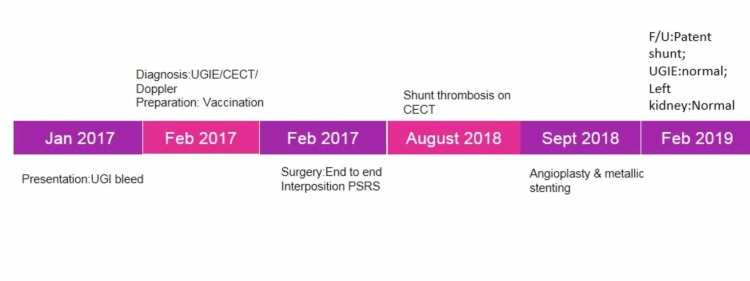
Timeline. UGIE: Upper gastrointestinal endoscopy; CECT: Contrast-enhanced computed tomography; UGI: Upper gastrointestinal; PSRS: Proximal splenorenal shunt.

Doppler ultrasound showed good flow in renal vein and shunt. There was no evidence of renal infarct. At two-year follow-up, UGI endoscopy was normal. The patient did not have any episode of UGI bleed postoperatively. Doppler ultrasound during further follow-up has confirmed patency of the shunt. There was no evidence of renal infarct and the size of the left kidney was normal. The timeline of events is shown in Figure 7.

## Discussion

Renal vein anomalies have been reported in the literature [2-7]. A thorough knowledge of the different anomalies is important as these anomalies may make it technically difficult or may preclude performance of splenorenal shunts. They may also be associated with increased risk of shunt failure [5]. Modification of surgical technique is required in such cases for an effective portal decompression without causing renal venous hypertension. The most common anomalies associated with LRV include circum-aortic double renal vein, retro aortic renal vein and late joining of the LRV tributaries [2]. Anomalous renal veins in general are more common on right side (38% cases) [3], while circum-aortic and retro-aortic renal veins are more common on left side. Retro-aortic LRV is reported in 1.8-3.4% cases in which there is a single LRV in retro-aortic position before it enters inferior vena cava (IVC) [4]. The normal LRV has a greater length and is more superficial in position in comparison to retro-aortic LRV. Retroaortic LRV has a restricted mobility which reduces the advantage of its greater length. Also compression on left renal vein due to its retro-aortic position may cause renal venous hypertension. A circum-aortic left renal vein or “renal collar” is seen in 5% of cases [5]. In these cases, the LRV is double, one vein passes posterior, and the other anterior to aorta before joining IVC. The other rare anomalies detected in various radiological and anatomical studies include late joining of the tributaries from hilum to form a short LRV near aorta, an oblique course of LRV with its joining at inferior vena cava at a lower level than usual and the gonadal or lumbar veins joining the branches instead of the main LRV [2, 3, 5-7].

To the best of our knowledge, the anomaly in LRV that we report has not appeared in the literature. In addition, this is the second report of LRV anomaly in NCPH [4].

There is scanty information in the literature about the technical difficulties associated with performance of PSRS in the presence of LRV anomalies [4]. One case of extrahepatic portal vein obstruction (EHPVO) with retro-aortic LRV detected preoperatively in imaging has been reported [4]. In this report, PSRS was successfully performed by dissecting greater length of splenic vein from pancreas, ligation of small pancreatic branches to the splenic vein and complete mobilisation of LRV from left margin of aorta to the renal hilum and ligation of left gonadal and adrenal veins [5]. In our case, we were not able to identify the LRV anomaly in the preoperative CECT because of an improperly phased CT.

In another report it has been suggested that an abnormal LRV does not pose difficulty during construction of Warren’s shunt, a selective shunt [8]. As PSRS aims at a more complete portal decompression (essential in cases with NCPH, especially EHPVO), an adequately mobilised LRV with a favourable anatomy is essential not only to ensure adequate portal decompression but also to avoid renal venous hypertension. Graft interposition shunt is a good option when one encounters such rare difficult anatomy. Primary-assisted patency rate and clinical response is reasonably good in this case. In our case, an interposition shunt with 8 mm PTFE graft could be constructed without much technical difficulty and there was no evidence of renal venous hypertension or recurrence of symptoms of portal hypertension during follow-up.

## Conclusions

This type of renal vein anomaly does not preclude performance of PSRS with suitable modifications. A high index of suspicion is required to detect them preoperatively to avoid technical difficulties and to plan modifications of PSRS. Interposition end-to-end graft proximal splenorenal shunt is a valid option with good primary-assisted patency rate and clinical outcome.
